# Distinct Properties of Hexameric but Functionally Conserved *Mycobacterium tuberculosis* Transcription-Repair Coupling Factor

**DOI:** 10.1371/journal.pone.0019131

**Published:** 2011-04-29

**Authors:** Swayam Prabha, Desirazu N. Rao, Valakunja Nagaraja

**Affiliations:** 1 Department of Biochemistry, Indian Institute of Science, Bangalore, India; 2 Department of Microbiology and Cell Biology, Indian Institute of Science, Bangalore, India; New England Biolabs, Inc., United States of America

## Abstract

Transcription coupled nucleotide excision repair (TC-NER) is involved in correcting UV-induced damage and other road-blocks encountered in the transcribed strand. Mutation frequency decline (Mfd) is a transcription repair coupling factor, involved in repair of template strand during transcription. Mfd from *M. tuberculosis* (MtbMfd) is 1234 amino-acids long harboring characteristic modules for different activities. Mtb*mfd* complemented *Escherichia coli mfd* (Eco*mfd*) deficient strain, enhanced survival of UV irradiated cells and increased the road-block repression *in vivo*. The protein exhibited ATPase activity, which was stimulated ∼1.5-fold in the presence of DNA. While the C-terminal domain (CTD) comprising amino acids 630 to 1234 showed ∼2-fold elevated ATPase activity than MtbMfd, the N-terminal domain (NTD) containing the first 433 amino acid residues was able to bind ATP but deficient in hydrolysis. Overexpression of NTD of MtbMfd led to growth defect and hypersensitivity to UV light. Deletion of 184 amino acids from the C-terminal end of MtbMfd (MfdΔC) increased the ATPase activity by ∼10-fold and correspondingly exhibited efficient translocation along DNA as compared to the MtbMfd and CTD. Surprisingly, MtbMfd was found to be distributed in monomer and hexamer forms both *in vivo* and *in vitro* and the monomer showed increased susceptibility to proteases compared to the hexamer. MfdΔC, on the other hand, was predominantly monomeric in solution implicating the extreme C-terminal region in oligomerization of the protein. Thus, although the MtbMfd resembles EcoMfd in many of its reaction characteristics, some of its hitherto unknown distinct properties hint at its species specific role in mycobacteria during transcription-coupled repair.

## Introduction

DNA is a dynamic molecule and is constantly exposed to various types of damaging agents such as mutagenic chemicals, radiation and reactive oxygen. A number of DNA repair systems exist which specialize in the repair of certain types of damage. Nucleotide excision repair (NER) is a highly conserved pathway involved in repair of a wide variety of structurally unrelated DNA lesions [1]. One of the well characterized NER systems is the UvrABC nuclease from *E. coli* [2], [3]. NER consists of two related sub-pathways; global genomic repair (GGR), which removes lesions from the overall genome, and transcription coupled repair (TCR), which removes lesions from the transcribed strand of active genes [4]–[6]. Bulky DNA lesions such as cyclo pyrimidine photodimers (CPD) induced by UV irradiation block RNA polymerase during transcription [6]. In bacteria a product of *mfd* called transcription repair coupling factor (TRCF) or Mfd protein is required for TCR [7]–[9]. Bacterial Mfd interacts with the stalled RNA polymerase, displaces it from the DNA and recruits NER proteins at the site of damage [10], [11]. Mfd thus clears the steric hindrance from the site of damage and loads UvrA protein, resulting in ∼10-fold faster repair of the transcribed strand compared to the non-transcribed strand for similar kind of lesions [12]. In addition, Mfd rescues arrested or backtracked transcription elongation complexes by promoting forward translocation of RNA polymerase in ATP dependent manner leading to productive elongation [13]. Additionally, Mfd can release the RNA polymerase when the enzyme cannot continue elongation [13]. Apart from DNA repair, Mfd has other physiological roles in regulation of gene expression, including carbon catabolite repression in *Bacillus subtilis* [14] and transcription termination by bacteriophage HK022 Nun protein [15]. A key role for Mfd as an enhancer of UvrA turnover in *E. coli* cells has also been recently demonstrated [16].

The well characterized Mfd from *E. coli* is a 130 kDa monomeric protein having modular architecture specialized for different functions [8]. The N-terminal domain (NTD) shares a high degree of structural homology with UvrB protein of NER pathway [17], [18]. The NTD is known to interact with UvrA protein, which is molecular matchmaker of NER pathway, and this interaction is responsible for enhanced rates of repair [8]. The central portion of Mfd consists of RNA polymerase interacting domain (RID) which binds to β subunit of RNA polymerase [13], [17]. The C-terminal domain (CTD) of Mfd harbors seven signature motifs of super-family 2 helicases including ATPase motifs. In addition, CTD contains a TRG motif (translocation in RecG) required for translocation along the DNA. TRG motif as the name implies, is highly homologous to RecG protein, which is known to be involved in branch migration of Holliday junctions during recombination [19], [20].

Pathogenic bacteria continuously encounter multiple forms of stress in their hostile environments, which leads to DNA damage. Genes involved in DNA repair and recombination may play an important role in the virulence of pathogenic organisms [21]. *M. tuberculosis* is a gram positive, acid fast bacterium and one of the most formidable human pathogen. DNA repair pathways in mycobacteria appear to be crucial for their survival at different stages of infection [22]. Sequencing of *M. tuberculosis* genome revealed the presence of NER associated genes including a putative *mfd*. In this work, we describe the functional characterization of MtbMfd and discuss its unusual properties. This is the first detailed analysis of the biochemical properties of Mfd from actinomycetes and more importantly from a human pathogen.

## Results

### Cloning, expression and functional characterization of MtbMfd

**Figure 1:**
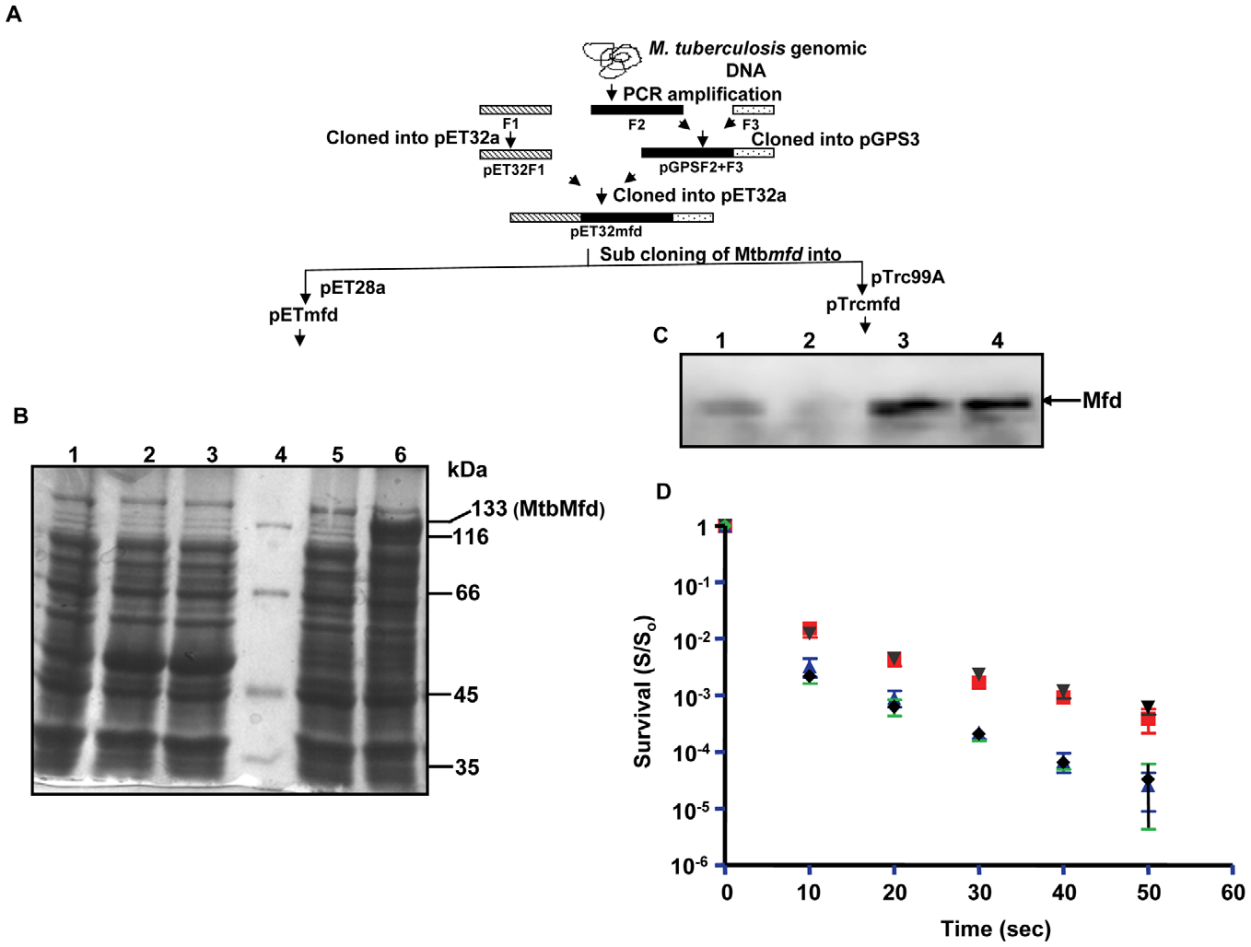
Cloning, expression and functional analysis of *M. tuberculosis mfd (Mtbmfd)*. **A**. Schematic showing the strategy used for cloning of *Mtbmfd*; F1, F2 & F3 represents three fragments of *Mtbmfd* obtained by PCR amplification from genomic DNA of *M. tuberculosis* by using set of specific primers. F1 was cloned into pET32a in MscI-KpnI sites and F2 was cloned into pGPS3 vector using KpnI-BsrB1 sites. F2+F3 fragment was obtained by ligation of F3 into PGPS3 containing F2 using BsrBI-HindIII sites. A 2.4 kb fragment containing F2+F3 was released using KpnI-HindIII sites from pGPS3 clone and ligated into pET32a F1 clone to obtain full length Mtb*mfd* gene. Sub cloning of Mtb*mfd* in pET28a and pTrc99A vectors were used for overexpression and *in vivo* assays respectively. **B**. SDS-PAGE analysis of overexpression of His-tagged MtbMfd in *E. coli* expression strain (Tuner) using pETmfd construct. Lane 1, total cell extracts of Tuner cells; lane 2, total cell extracts of uninduced Tuner cells carrying pET28a vector alone; lane 3, induced cell extracts of Tuner cells harboring pET28a vector; lane 4, protein molecular weight marker; lane 5, total cell extracts of uninduced Tuner cells carrying pETmfd construct and lane 6, total cell extract of induced cell extracts (0.3 mM IPTG) of Tuner cells carrying pETmfd construct. **C**. Western blot analysis using anti-MtbMfd polyclonal antibody for expression of Mfd in *E. coli* stains used for complementation studies; lane 1, AB1157; lane 2, UNCNOMFD; lane 3, pTrcmfd in presence of 0.5 mM of IPTG and lane 4, pTrcmfd. **D**. Effect of UV on survival (S/S_0_) of *E. coli* strains; AB1157 (red, ▪); UNCNOMFD (blue, ▴); pTrcmfd (brown,▾) and pTrc99A (black,♦). Each value represents the average from three independent experiments. (Survival = S/S_0_; where S_0_ = number of bacterial colonies obtained without UV irradiation and S = number of bacterial colonies obtained after UV irradiation). (AB1157, *E. coli* wild-type strain; UNCNOMFD, *mfd* deficient stain of *E. coli*; pTrcmfd, UNCNOMFD transformed with Mtb*mfd* construct and pTrc99A, UNCNOMFD transformed with pTrc99A vector alone).

Genome analysis of *M. tuberculosis* revealed that MtbMfd is 1234 amino acids long encoded by 3.7 Kb DNA fragment. Cloning of the *Mtbmfd* was carried out by reconstructing the full length gene from three PCR amplified fragments using gene specific primers (Table 1) and genomic DNA as a template. The strategy is depicted in Figure 1A and details are described in Materials and Methods. Mfd cloned under the control of T7 promoter (pETmfd) was used for overexpression and purification, whereas the gene cloned under trc promoter (pTrcmfd) was used for *in vivo* assays (Fig. 1A). Heterologous expression of MtbMfd protein was achieved in *E. coli* strain BL21 (pLysS) TUNER as N-terminal histidine tagged protein using pETmfd construct. The overexpressed protein had the molecular mass of ∼133 kDa (Fig. 1B, lane 6) corresponding to the theoretical molecular weight of MtbMfd calculated from amino acid sequence. Whereas, no such protein was seen in vector control cell lysate (Fig. 1B, lanes 1–3) as well in uninduced cell lysate (Fig. 1B, lane 5).

**Table 1:** List of oligonucleotides (primers) used in this study.

PF1	5′GTGGCCATATGACCGCACCGGGGC3′
PR1	5′CAAGGTACCGGTGCCAGGCGCGAC3′
PF2	5′CGA GGTACCGCACACCGCGTG3′
PR2	5′CCCCGCTCGACGATCAAAGCT TTGGC3′
PF3	5′CCGAGCGGGCCGATACCTTC3′
PR3	5′CCGAAGCTTCACGGTTGTCGCTCC3′
PRdel	5′CATAAGCTTCAGGGTTCTTCGGCGGTCCT3′
WbF	5′CAGCTGTCGCGGTATGTCGGC3′
WbM	5′CTCCTCAGCGACCACCACCAG3′
WbR	5′GTCCAGAGTCTTGGCGTTGGAGAT3′

One of the *mfd* mutants of *E. coli* (UNCNOMFD) was shown to confer moderate ultraviolet sensitivity to the *E. coli* cells [8]. To determine the cellular function of MtbMfd, *in vivo* complementation assay was carried out using the above mentioned *mfd* deficient strain of *E. coli*. The effect of UV irradiation on survival (S/So) of wild-type *E. coli* (AB1157), UNCNOMFD and UNCNOMFD transformed with Mtb*mfd* construct (pTrcmfd) was determined as described in Materials and Methods. First, the expression of Mfd protein in *E. coli* was verified by western blot analysis using anti-MtbMfd polyclonal antibodies. UNCNOMFD strain did not express any detectable level of Mfd protein (Fig. 1C, lane 2) compared to AB1157 (Fig. 1C, lane 1). When UNCNOMFD was transformed with pTrcmfd construct, a considerable amount of MtbMfd protein was expressed (Fig. 1C, lane 4) which increased further upon addition of 0.5 mM of IPTG (Fig. 1C, lane 3). Next, AB1157, UNCNOMFD and pTrcmfd cells were exposed to UV for varied time and survival was determined. A ∼10 fold decrease in survival was observed in UNCNOMFD compared to AB1157 after irradiation. When UNCNOMFD strain was complemented with plasmid pTrcmfd, the survival was restored to the wild-type level (Fig. 1D) indicating that Mtb*mfd* complements *E. coli* counterpart in *mfd* deficient strain.

### MtbMfd increases the road-block repression *in vivo*

**Figure 2:**
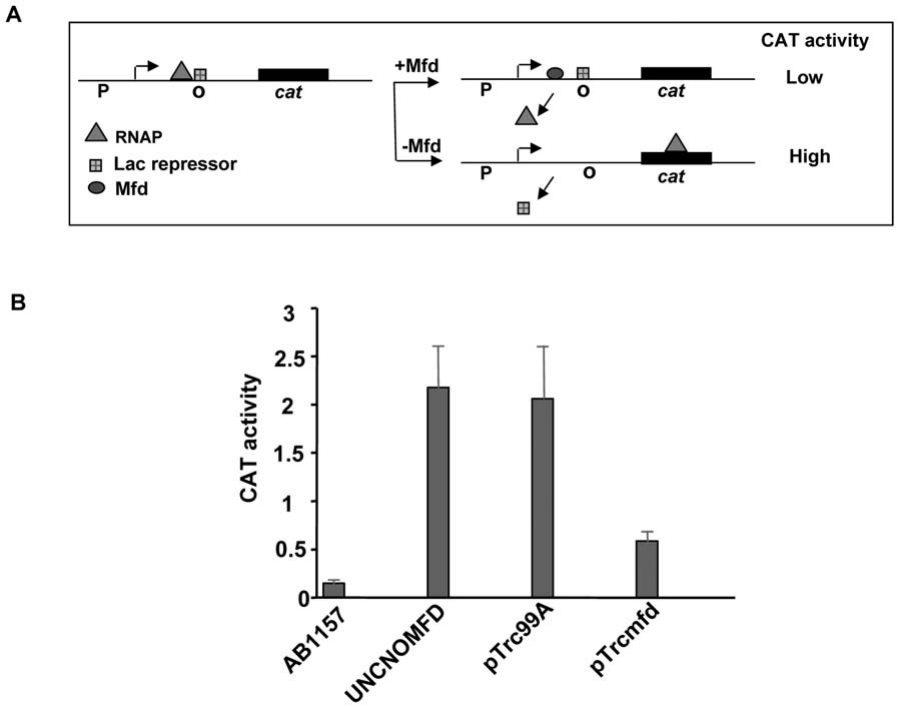
*In vivo* reporter assay for MtbMfd function. **A**. Schematic representation of the chloramphenicol acetyl transferase (cat) gene construct used for the assay. P is promoter site and O is the operator binding site for binding of Lac repressor. **B**. Scoring of CAT activity in the cell extracts of different *E. coli* strain as described in Materials and Methods; AB1157 (*mfd*+) or UNCNOMFD (*mfd*−), pTrcmfd (*mfd*+) and pTrc99A (*mfd*−). Cells were transformed with the roadblock repression reporter vector pRCBCAT1 and with plasmids encoding MtbMfd and Lac repressor. CAT activities shown are the average of three independent experiments and are expressed in units of nmol of chloramphenicol acetylated/min/mg of protein.

Transcription elongation complexes tend to pause or stall when they encounter a protein road-block or DNA damage on the template strand, reducing the transcription of downstream sequences. This has been observed *in vivo*, where formation of the protein road-blocks influences regulation of gene expression as in the case of the carbon catabolite repression of *hut* and *gnt* operons of *Bacillus subtilis* [14]. An *in vitro* assay has been developed in which expression of a reporter gene could be monitored in presence and absence of Mfd when RNA polymerase is stalled by Lac repressor [20]. The rationale behind this assay is that when operator site is occupied by Lac repressor, it blocks RNA polymerase engaged in transcription elongation. Mfd recognizes stalled RNA polymerase and removes it from the site of transcription resulting in lower *cat* transcription and CAT activity. However, in the absence of Mfd, paused RNA polymerase leads to high level of transcription as well as CAT activity after dissociation of the Lac repressor from the operator. To further confirm the functionality of MtbMfd, road-block reporter assays were carried out using pRCB-CAT1 construct [20] (Fig. 2A). AB1157 (*mfd+*) showed lowest CAT activity followed by UNCNOMFD cells complemented with pTrcmfd construct (Mtb*mfd*+) compared to UNCNOMFD (*mfd−*) and vector control, pTrc99A (*mfd*−) (Fig. 2B). These results suggested that MtbMfd complements *E. coli* counterpart and when MtbMfd was present in the system, road-block repression increased significantly. From these results it is apparent that MtbMfd interacts with *E. coli* RNA polymerase leading to its dissociation from the site of transcription.

### Overexpression and purification of MtbMfd proteins

**Figure 3:**
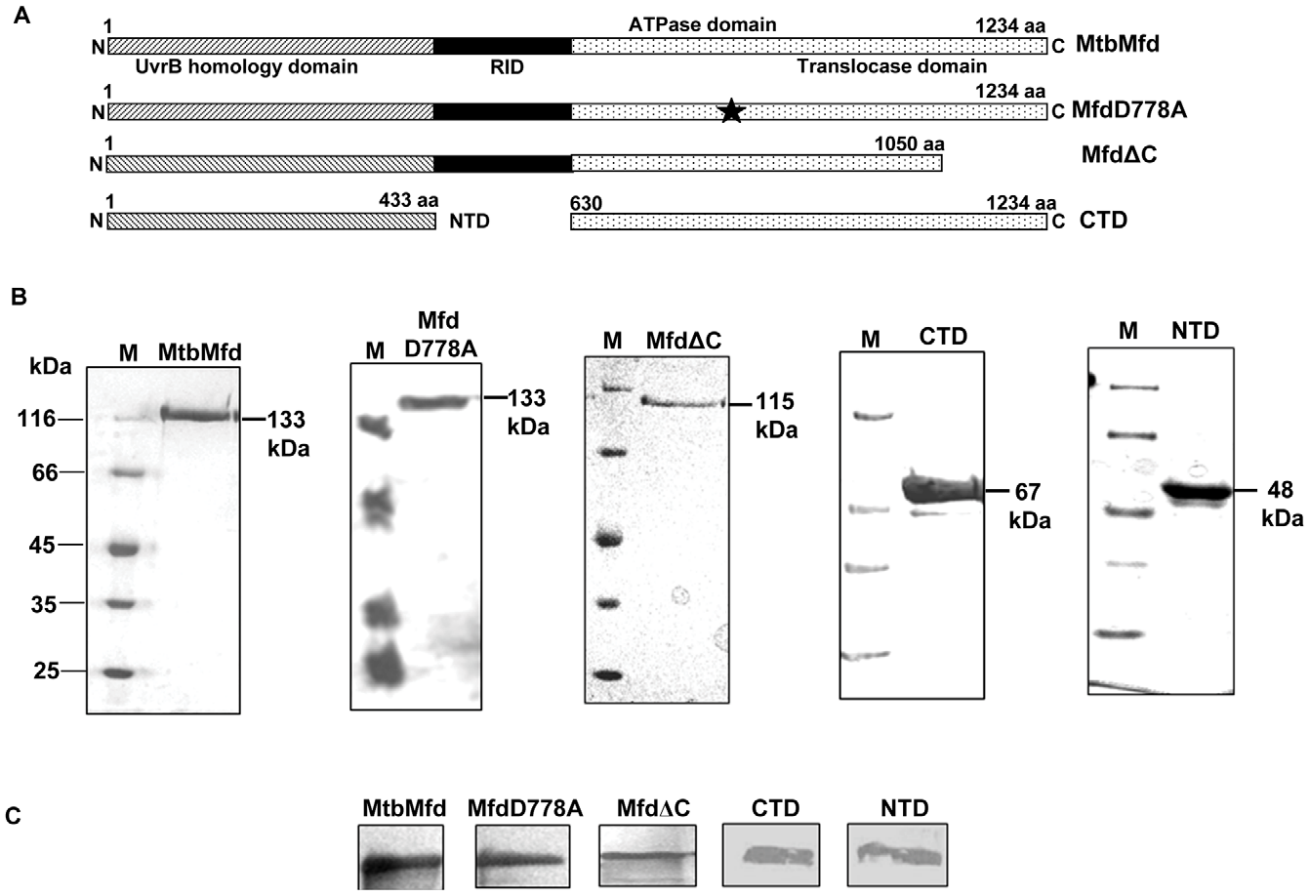
Purification and Western blot analysis of MtbMfd and its derivatives. **A**. Arrangement of conserved domains in the primary sequence of MtbMfd. It was generated by using SMART program for domain analysis as well as sequence alignment with EcoMfd. Nomenclature used here is similar to that of EcoMfd. Various constructs of MtbMfd generated for this study are shown in the diagram; full length MtbMfd (1–1234 aa), MfdD778A ((1–1234 aa) where star represents the site of mutation in the ATPase domain, MfdΔC (1–1050 aa), NTD (1–433 aa) and CTD (630–1234 aa). **B**. SDS-PAGE analysis of purified full length MtbMfd and truncated proteins, M represents the protein molecular weight markers along with other purified proteins; full length MtbMfd (∼133 kDa); MfdD778A (∼133 kDa); MfdΔC (∼115 kDa); CTD (∼67 kDa) and NTD (∼48 kDa). **C**. Western blot analysis of purified MtbMfd protein and its derivatives using anti-Mfd polyclonal antibody raised in rabbit. Each experiment was done separately.

The homology between MtbMfd and EcoMfd is 38% over the entire length. The important domains and their linear organization along the sequence are conserved between the two proteins. MtbMfd (1234 aa) contains UvrB homology domain at the N terminal, a RNA polymerase interacting domain (RID) in the central part and ATPase and translocase domains in the C-terminal. The schematic representation in Fig. 3A depicts the full length and other constructs of MtbMfd generated for this study. In addition to full-length protein, a mutant MtbMfd, MfdD778A was generated by changing a single amino acid in Walker B motif of ATPase domain. A construct referred to as MfdΔC was generated which harbors amino acids 1–1050 but lacking the extreme 184 residues from the C-terminus. Another construct referred to as CTD, spanning amino acids 630–1234 having intact C-terminal region was generated. A third construct named as NTD comprising amino acids 1–433 was also generated. Mfd and its variants were purified to apparent homogeneity as described in Materials and Methods. Purified MtbMfd was subjected to mass spectrometry analysis to confirm the authenticity of the protein, and the result obtained matched with that of the theoretical amino acids sequence of the MtbMfd (data not shown). The purity of the proteins were checked on SDS-PAGE and the experimental molecular masses were in agreement with their predicted molecular weights (full length MtbMfd and MfdD778A are ∼133 kDa each, MfdΔC is ∼115 kDa, CTD is ∼67 kDa and NTD is ∼48 kDa) (Fig. 3B). Western blot analysis of the purified proteins using anti-MtbMfd polyclonal antibodies detected full length MtbMfd as well as its variants (Fig. 3C).

### Unusual behavior of MtbMfd in solution

**Figure 4:**
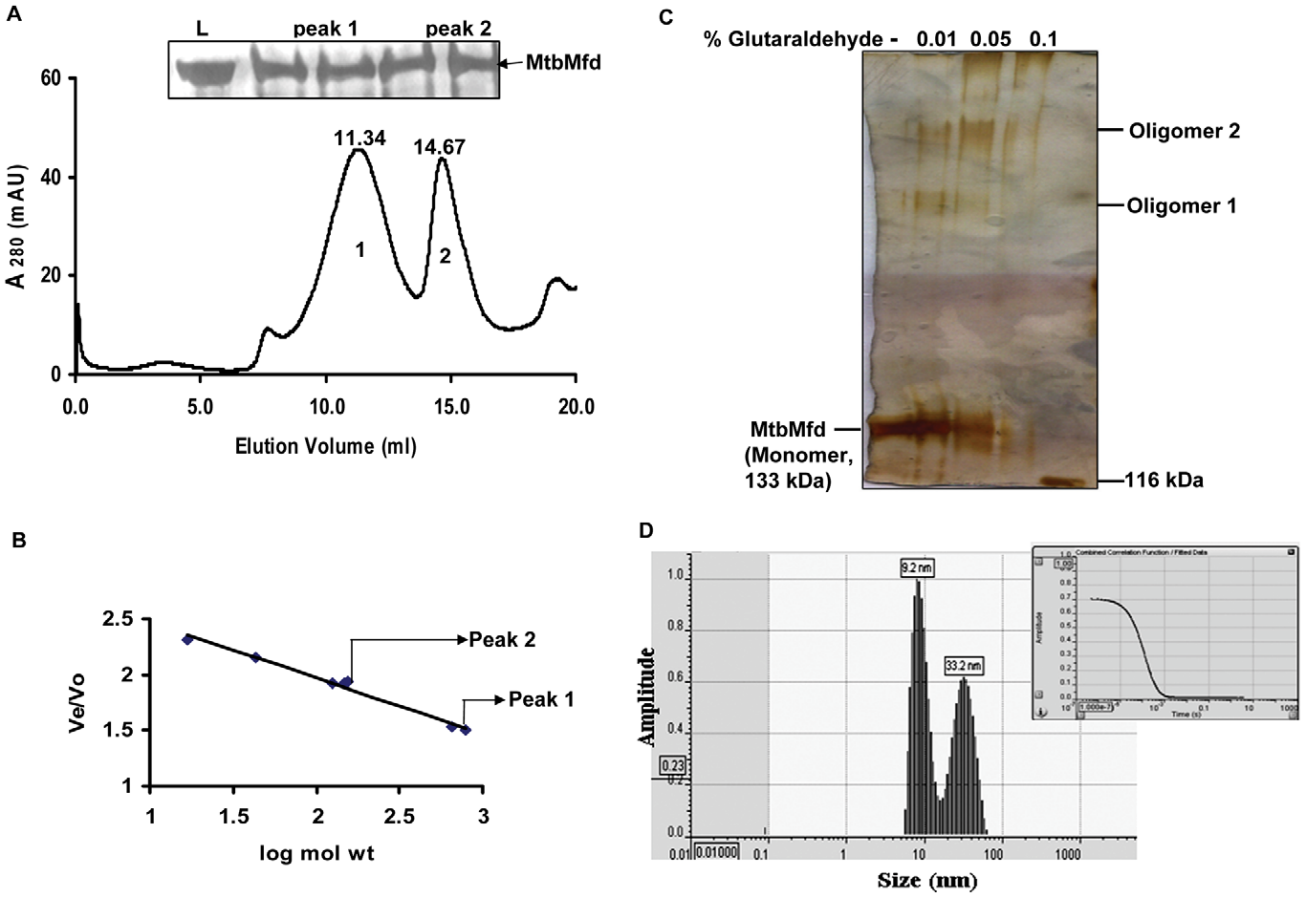
Determination of oligomeric status and molecular mass of MtbMfd under native conditions. **A**. Elution profile of MtbMfd (700 µg) using gel filtration chromatography where the two peaks are shown as peak 1 and peak 2 and numbers denoted above the peaks represent their retention volume. Inset of the graph is SDS-PAGE profile showing the presence of MtbMfd in both the eluted peaks (L represents the MtbMfd sample before gel filtration). **B**. Standard curve Ve/Vo *versus* the log of molecular weight which was derived from the elution profiles of the standard molecular weight markers (described in Materials and Methods). Ve corresponds to the elution volume of the protein and Vo representing the void volume of the column which was determined using Blue dextran. **C**. Chemical crosslinking of MtbMfd with increasing concentrations of glutaraldehyde (0–0.1%). Oligomers 1 & 2 represent the cross-linked species of MtbMfd in presence of glutaraldehyde. **D**. Dynamic light scattering profile of MtbMfd. Light scattering experiment was performed with 100 nM–1 µM of purified His-tagged MtbMfd in 10 mM Tris-Cl (pH 8.0) using DynaPro DLS instrument. Hydrodynamic radius was calculated by DYNAMICS v.3.3 software. The histogram represents the intensity distribution of MtbMfd sample and inset represents combined correlation function of fitted data. The R_H_ value of 9.2 nm corresponds to hexamer of MtbMfd and 33.2 nm corresponds to protein aggregate.

To determine the oligomeric status of MtbMfd, gel filtration analysis was carried out. Surprisingly, MtbMfd eluted as two peaks (Fig. 4A) at positions corresponding to a globular protein of ∼790 kDa (peak 1) and ∼130 kDa (peak 2) respectively (Fig. 4B). The presence of MtbMfd in both the peaks was confirmed by SDS-PAGE analysis (Inset of Fig. 4A, panel showing SDS-PAGE). The molecular weight of peak 1 and peak 2 corresponded to the hexamer and monomer size of MtbMfd, respectively. Chemical cross-linking of MtbMfd with glutaraldehyde was carried out to determine the oligomeric nature of the protein. Glutaraldehyde is a homo bifunctional cross-linking reagent that cross-links N-terminal primary amines of lysine residues, resulting in the formation of amidine cross-links between protein subunits. Glutaraldehyde treated MtbMfd migrated with a lower mobility than the monomer of MtbMfd (133 kDa). Two reduced mobility crosslinked products were observed on SDS-PAGE (oligomer 1 and 2, Fig. 4C). The majority of the crosslinked species was the slowest migrating species (oligomer 2) suggesting that the oligomer 1 form may be an intermediate product of cross linking reaction. The hydrodynamic radius (R_H_) of MtbMfd was calculated by dynamic light scattering experiments (DLS). 100 nM–1 µM of purified MtbMfd protein was subjected to DLS analysis and a R_H_ value of 9.2±1 nm obtained corresponded approximately to the hexamer of MtbMfd as estimated by comparison with typical globular proteins (Fig. 4D).

EcoMfd is known to be monomeric in nature [17]. In spite of its structural and functional similarity to the EcoMfd, the existence of MtbMfd in hexameric form prompted us to carry out further analysis to confirm the oligomeric nature of the protein. The behavior of the protein was analyzed by gel filtration chromatography under different conditions. Equimolar concentrations of MtbMfd (∼133 kDa) and thyroglobulin (669 kDa) were mixed and co injected into the column. The two peaks obtained were analyzed on SDS-PAGE and noticeably, the fraction corresponding to peak1 retained both MtbMfd and thyroglobulin whereas peak 2 retained only MtbMfd (Fig. S1A). Co-elution of hexameric MtbMfd with thyroglobulin and the elution of monomeric MtbMfd separately support the existence of two forms of MtbMfd in solution. To analyze the effect of protein concentration on oligomeric status of MtbMfd, two different concentrations of the protein were injected into the column. The distribution of the monomer and hexamer peaks of MtbMfd was different at different protein concentrations (Figs. S1B & C). High salt is known to disrupt the non specific aggregation of the proteins and therefore gel filtration was performed in presence of 500 mM NaCl. The profile of MtbMfd obtained was similar to that of 100 mM NaCl elution pattern indicating that the hexamer of MtbMfd is stable at higher ionic environment (Fig. S1D). Collectively, these experiments suggest that the existence of two forms of MtbMfd is concentration dependent and stable at high salt.

**Figure 5:**
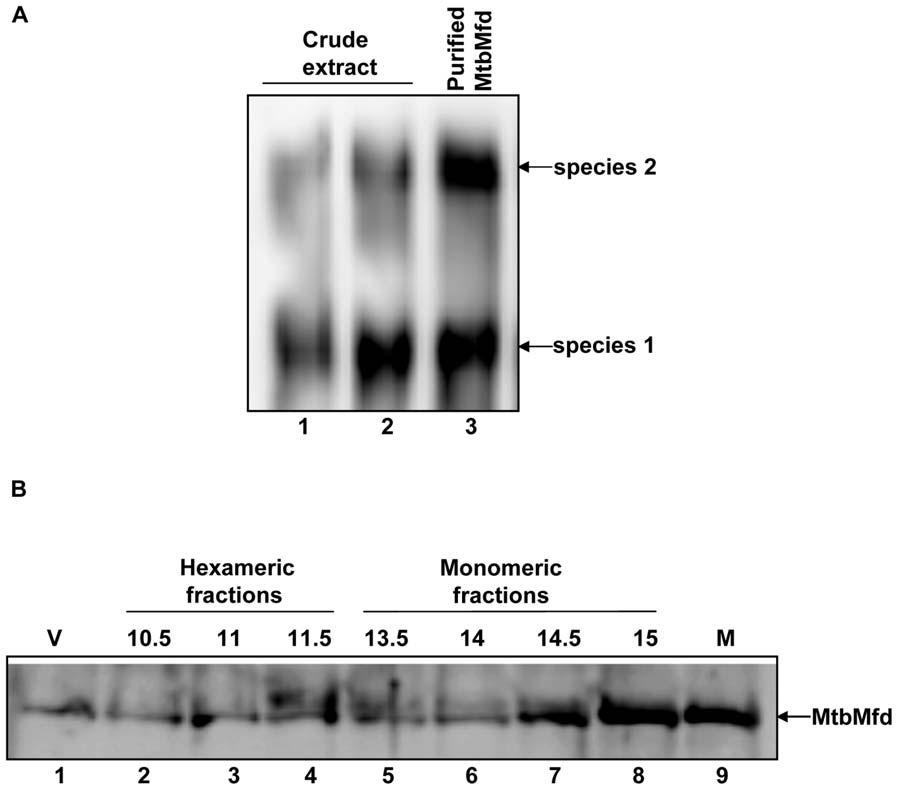
Determination of oligomeric status of MtbMfd *in vivo*. *M. tuberculosis Ra* strain was grown in 7H9 medium containing 10% ADC supplements and 0.05% tween 80 up to 0.7 OD. Crude cell lysate was prepared in Tris buffer pH 8.0 by sonication followed by ultracentrifugation at 100,000 g (S100). The supernatant was subjected to further analysis. **A**. Native-PAGE (6%) Western profile of cell free lysate of *M. tuberculosis H37Ra* in Tris-Glycine buffer pH 8.3 at room temperature, the blot was probed with anti-MtbMfd antibodies. Lanes 1 & 2, 25 and 50 µg of crude cell lysate of *M. tuberculosis H37Ra* and lane 3, purified MtbMfd protein. **B**. Crude cell lysate of *M. tuberculosis Ra* was subjected to gel filtration chromatography on superose 6 column and fractions were collected and analyzed on SDS-PAGE followed by Western blot using anti MtbMfd antibody. Lane 1, V corresponds to void volume fraction (protein complex or aggregates); lanes 2–4, 10.5, 11 and 11.5 ml fractions (hexamer of MtbMfd); lanes 5–8, 13.5,14,14.5 and 15 ml fractions (monomer of MtbMfd) and lane 9, purified MtbMfd used as a marker. Fractions 10.5–11.5 ml and 13.5–15 ml represents the hexamer and monomer species of MtbMfd present in the crude cell lysate.

In order to check whether oligomeric forms of MtbMfd exist *in vivo*, the following experiments were carried out using cell lysate of *M. tuberculosis H37Ra*. First, native-PAGE Western blot analysis was carried out with the crude cell lysate using anti-MtbMfd antibody and it was found that MtbMfd exists as predominantly in two forms in the cell (Fig. 5A). Second, cell lysate of *M. tuberculosis* was subjected to gel filtration chromatography under non-denaturing conditions (Fig. S2A) and eluted fractions were analyzed by Western blot using anti-MtbMfd antibody. The hexameric fractions eluted between 10 to11.5 ml while the monomeric MtbMfd was present in the 14 to15 ml fractions (Fig. 5B). Gel filtration chromatography of purified native MtbMfd (Fig. S2B) showed a similar profile as described for His-tagged MtbMfd.

**Figure 6:**
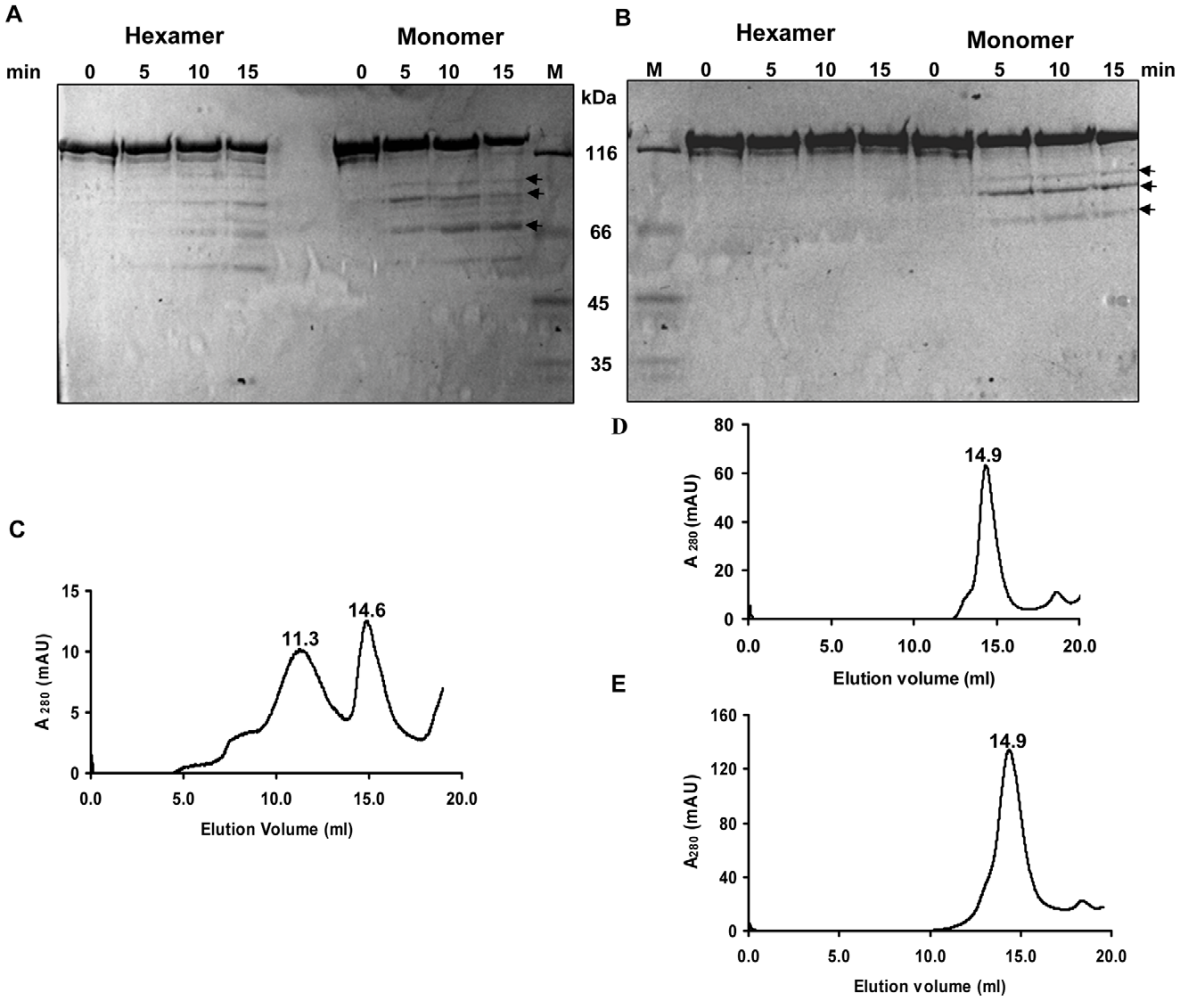
Limited proteolysis of MtbMfd and oligomeric status of full length MtbMfd versus MfdΔC. (A and B) Hexamer and monomer fractions obtained by gel filtration chromatography were subjected to protease digestion. SDS-PAGE showing the pattern of polypeptide fragments produced by **A**. trypsin and **B**. V8 protease digestion along with molecular weight markers. The time of digestion is indicated along the top of the gel. Gel filtration analysis of purified proteins. **C**. Full-length MtbMfd (250 µg). **D**. MfdΔC (250 µg). **E**. MfdΔC (600 µg).

Limited proteolysis is often employed to determine the domainal organization, stability and conformational changes within the protein. The hexamer and monomer fractions of MtbMfd obtained by gel filtration chromatography were subjected to limited digestion by trypsin and V8 protease to further explore the characteristics of the two species of the protein. Trypsin cleaves peptide bonds exclusively at C-terminal of arginine and lysine residues and V8 protease cleaves on the carboxyl side of glutamic acid. The digestion with trypsin gave multiple bands with both the forms of MtbMfd in a time dependent manner but monomeric fraction showed more sensitivity (Fig. 6A). Similarly, V8 protease digestion showed that the hexameric fraction was significantly more resistant compared to the monomer fraction (Fig. 6B). To assess the functional significance of oligomerization of MtbMfd, ATPase assay was carried out with the hexamer and monomer fractions of MtbMfd after separating them by size exclusion chromatography. It was found that both the forms of MtbMfd were able to hydrolyze ATP. However, the specific ATPase activity of monomer (156.78 pmoles ATP hydrolyzed/min/µg of protein) was ∼3-fold higher compared to the hexamer (58.1 pmoles of ATP hydrolyzed/min/µg of protein) (Fig. S3). Since both the forms of MtbMfd showed ATPase activity, next we considered possible ligand mediated transition between these forms. Gel filtration was carried out in the presence of ATP and DNA and it was observed that the elution profile of MtbMfd did not alter in presence of these two substrates (Fig. S4 A–E).

In addition, the oligomeric status of the purified individual domains of MtbMfd (NTD and CTD) was determined and they were found to be monomeric in nature (data not shown). When MfdΔC was subjected to gel filtration chromatography and compared with full-length MtbMfd (Fig. 6C), surprisingly a majority of MfdΔC eluted as a monomer even at higher concentrations (Fig. 6D and E). These results indicate that the extreme C-terminus region could be important for MtbMfd to acquire an oligomeric form.

### Characterization of ATPase activity of MtbMfd and its derivatives

**Figure 7:**
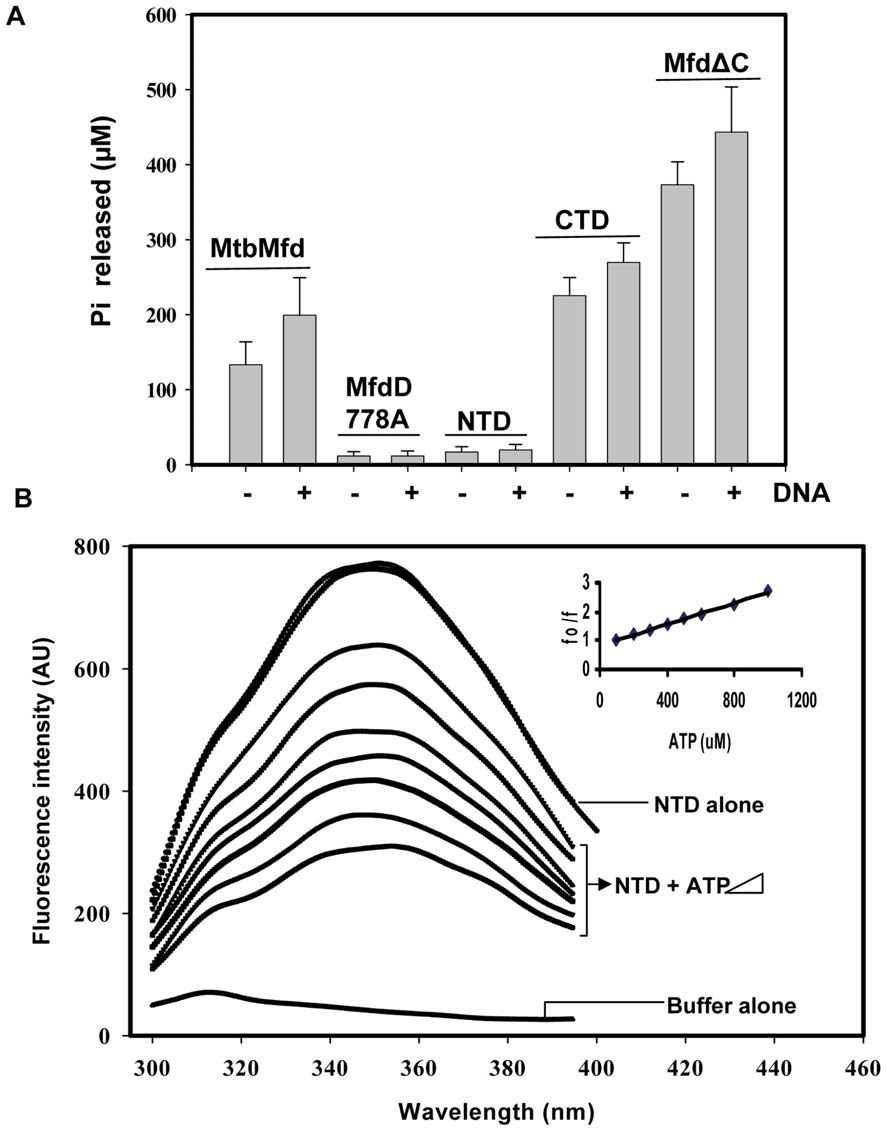
ATP hydrolysis by MtdMfd and its derivatives. **A**. Comparison of ATPase activity by MtbMfd and its derivatives. Proteins (250 nM each) were incubated with ATP in reaction buffer both in absence and presence of DNA as described in Materials and Methods. Results shown here is an average of the three experiments. **B**. NTD-ATP interaction study; 1 µM of NTD was incubated in buffer without MgCl_2_ with increasing concentration of ATP (0–1 mM) at 25°C and fluorescence emission spectra were monitored. Graph depicts the quenching of intrinsic fluorescence (arbitrary units) in presence ATP and inset represents Stern-Volmer plot used for calculation of binding constant (Ksv) for ATP.

Mfd hydrolyses ATP in order to displace RNA polymerase from the site of damage [13]. It possesses a typical ATPase active site having Walker A and B motifs towards its C-terminal region. To analyze the kinetics of ATP hydrolysis of MtbMfd and its truncated proteins, reactions were carried out using radiolabeled ATP as a tracer along with unlabeled ATP. Wild-type MtbMfd protein exhibited ATPase activity which was stimulated ∼1.5- fold in presence of dsDNA. The mutant MtbMfd (D778A) which harbors mutation in one of the key residues of Walker B motif of the ATPase domain, showed negligible ATPase activity indicating the importance of residue D778 for ATP hydrolysis (Fig. 7A). The kinetic parameters for MtbMfd were determined under steady state conditions. The turnover number (k_cat_) of 3.3±0.2 min^−1^ and a K_m_ (ATP) of 1.1±0.3 mM obtained at pH 8.0 and 37°C are comparable to those for EcoMfd. However, the turnover number for EcoMfd ATPase reported by different groups varies from 2.3–8.0 min^−1^ [8], [23], [24]. It can be seen from Table 2 that while the turnover number of MtbMfd did not significantly increase in the presence of DNA, the affinity for ATP (K_m_) increased ∼2 fold. Next, the turnover number of ATP hydrolysis for the CTD of MtbMfd was determined to be 5.2±0.5 min^−1^ (Table 2). In contrast, a higher level of ATPase activity was reported for CTD of EcoMfd (turnover number, 190 min^−1^) [24]. The huge difference in the rate of ATP hydrolysis between the two CTD proteins could account for their differences in translocase activity (see the later section). In the presence of DNA, the affinity of MtbMfd CTD towards the ATP increased by ∼2 fold (Table 2) similar to the full length MtbMfd suggesting that the deletion of first 600 residues did not alter the DNA binding properties. In contrast to full length MtbMfd, MfdΔC showed robust ATPase activity with a ∼10 fold higher turnover number for ATP hydrolysis (27.6±1.2 min^1^) (Table 2). This result is similar to the one obtained for the *E. coli* MfdΔC [23], implicating an auto regulatory function for the extreme C-terminus of MtbMfd. Unlike the classical ATPase motif which is present at the C-terminal of Mfd, an additional RecA like domain is located at the N-terminal of Mfd which resembles the one found in UvrB protein [18]. In order to check whether the purified NTD of MtbMfd can perform ATP binding and hydrolysis, assays were carried out as described in Materials and Methods. Notably, fluorescence quenching studies demonstrate that the NTD of MtbMfd binds ATP in a concentration dependent manner. Quenching of intrinsic fluorescence was observed in presence of ATP with Ksv constant of 526 µM (Fig. 7B). However, NTD was deficient in ATP hydrolysis (Fig. 7A). Previous studies with EcoMfd revealed that the degenerate ATPase motif in its NTD to be deficient for the nucleotide binding and hydrolysis [18].

**Table 2:** Kinetic parameters of *M. tuberculosis* Mfd ATPase.

Proteins	*k*_cat_ (min^−1^)		*K*_m_ (mM)	
	−DNA	+DNA	−DNA	+DNA
MtbMfd	3.3±0.7	5.3±0.5	1.1±0.3	0.6±0.08
CTD	5.2±0.8	8.3±0.4	1.3±0.2	0.6±0.2
MfdΔC	27.6±1.2	43.7±2	0.65±0.2	0.32±0.1

### Translocation of MtbMfd along DNA

**Figure 8:**
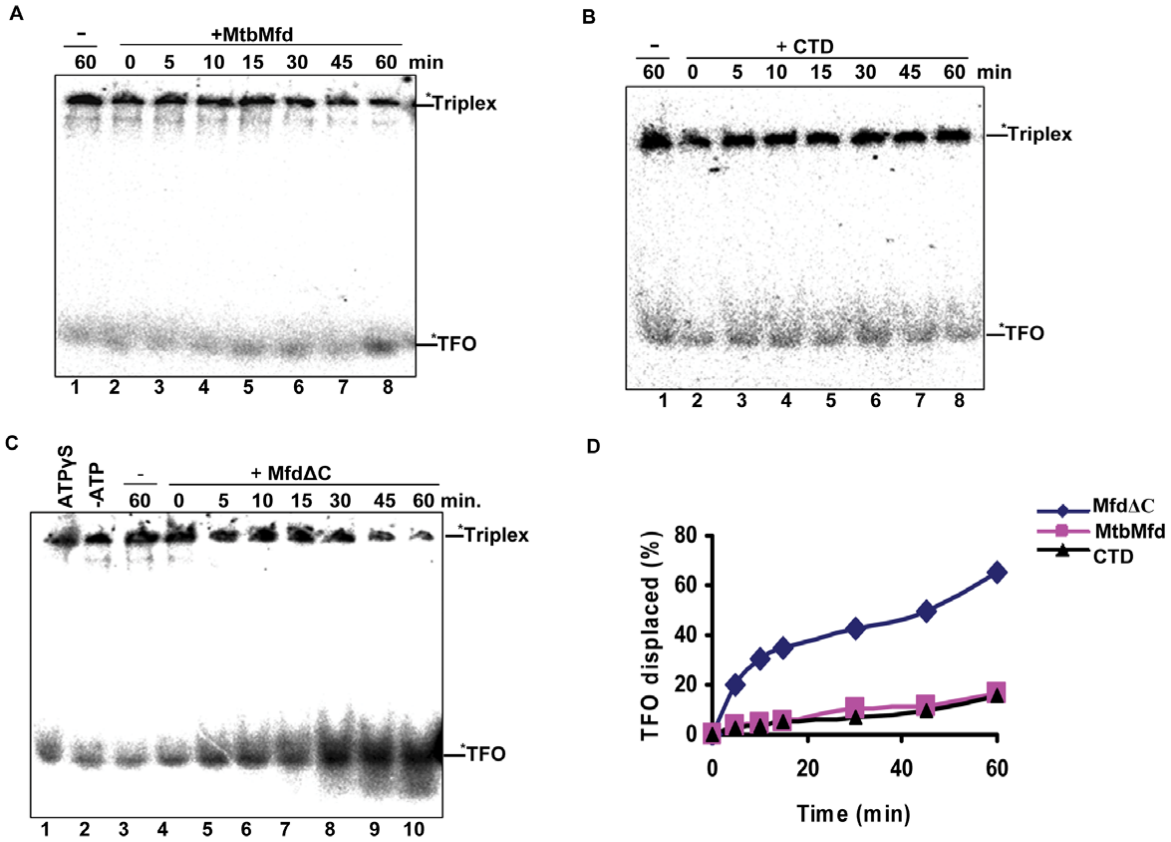
Triplex forming oligonucleotide (TFO) displacement or translocase assay for MtbMfd. Triplex DNA was incubated with 250 nM MtdMfd or its derivatives. Reactions were initiated by the addition of 2 mM ATP and 10 mM MgCl_2_ at 20°C. Aliquots were removed at the indicated time points, quenched and product analyzed on 7% polyacrylamide gel electrophoresis at 4°C. **A**. In presence of wild-type MtbMfd; lane 1, without protein; lanes, 2–8 in presence of MtbMfd. **B**. In presence of CTD; lane 1, without protein; lanes, 2–8 in presence of CTD. **C**. MfdΔC (250 nM) was incubated with triplex substrate in presence of ATPγS (lane 1); in absence of ATP (lane 2); and in presence of ATP (lanes, 3–10) 2 mM each and scored for the activity at 20°C. Lane 3, without MfdΔC. Time is indicated on the top of the each gel. Triplex substrate and ssDNA product is denoted on the right side of the gel and star indicates the radiolabeling. **D**. Quantitation of data obtained in A, B & C and the graph shows the percentage of TFO displaced from triplex substrate at each time point by full length MtbMfd (▪), MfdΔC (♦) and CTD (▴).

Mfd belongs to super-family 2 (SF2) helicases and is known to translocate along the DNA to displace RNA polymerase in an ATP dependent manner [13]. The translocase activity of MtbMfd was measured on linear triplex DNA substrate by carrying out TFO (Triplex Forming Oligonucleotide) displacement assay described previously for EcoMfd [23]. The triplex linear DNA was separately incubated with MtbMfd, CTD and MfdΔC and the displacement of radiolabeled TFO was monitored on the polyacrylamide gel. MtbMfd did not exhibit significant translocase activity under these assay conditions (Fig. 8A, lanes 2–8). This is similar to the data obtained with EcoMfd [23]. Interestingly, the CTD of MtbMfd did not show detectable level of translocase activity (Fig. 8B, lanes 2–8) unlike the CTD of *E. coli* which was shown to efficiently translocate along DNA possibly because of its high ATPase activity [24]. Unlike CTD, MfdΔC from *M. tuberculosis* exhibited efficient ATPase activity and also showed robust translocase activity (Fig. 8C, lanes 4–10) in an ATP dependent manner. About 80% of the ssDNA was displaced from the triplex substrate (Fig. 8D) and these results are similar to those obtained for *E. coli* MfdΔC [23]. In the absence of ATP or in the presence of a non-hydrolysable form of ATP (ATPγS) in the reaction, the translocase activity of MtbMfdΔC was found to be negligible (Fig. 8C, lanes 1 & 2). These results provide a direct correlation between translocase and ATPase activity of MtbMfd, and suggest the dependence of the former on the later reaction. Although Mfd possesses helicase motifs in the C-terminal region, the purified MtbMfd did not appear to unwind DNA: DNA hybrids (data not shown) reiterating the notion that all translocases do not necessarily function as helicases.

### NTD overexpression affects cellular function

**Figure 9:**
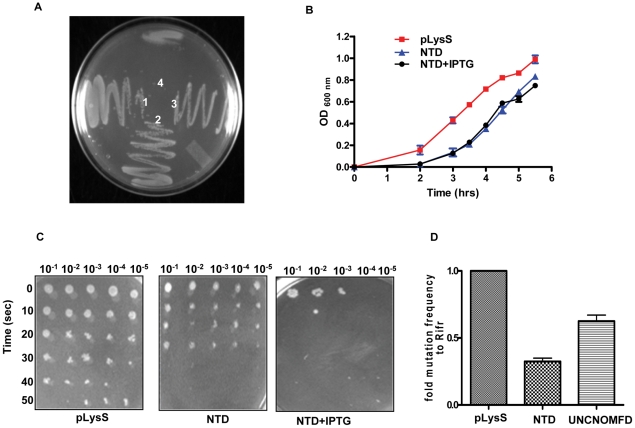
Effect of overexpression of NTD on cell physiology and growth. **A**. Growth characteristic of wild-type and NTD expressing *E. coli* cells. 1 pLysS alone; 2 pLysS transformed with pET14b; 3 uninduced pLysS cells carrying NTD construct and, 4 induced (0.3 mM IPTG) pLysS cells carrying NTD. **B**. Growth kinetics of wild-type and NTD expressing cells in liquid medium (± IPTG). **C**. Effect of UV irradiation on the survival of NTD expressing cells. pLysS cells carrying NTD and pET14b vector alone were grown in media containing ampicillin and chloramphenicol up to 0.6 OD. Equal number of cells were irradiated with UV light (1 J/m^2^) at different times and several dilutions of each cells were spotted on the LB agar plate containing appropriate antibiotics and incubated overnight at 37°C. The entire procedure after UV treatment was carried out in dark. **D**. Effect of NTD expression on the rate of spontaneous mutation frequency in *E. coli* cells. Fold change in mutation frequency was determined as described in Materials and Methods. Each experiment was performed at least three times. (UNCNOMFD, *mfd* deficient strain of *E. coli*, was taken as control).

Sequence comparisons of NTD of Mfd and UvrB showed that NTD retains intact UvrA interacting domain of UvrB and probably recruits UvrA during TCR [18]. Overexpression of NTD of MtbMfd in *E. coli* resulted in a delayed growth phenotype. To explore this further, growth of wild-type and the NTD expressing cells was monitored on solid and liquid medium. The NTD expressing cells showed growth defects on solid medium (Fig. 9A) whereas delayed growth phenotype was observed in liquid medium (Fig. 9B). On the other hand, no such defects were observed when other truncated MtbMfd proteins viz MfdΔC, CTD and RID were overexpressed (data not shown). Since NTD harbors an UvrA interacting domain, when it is expressed it may sequester the cellular pool of UvrA leading to dominant negative phenotype. When UV survival assays were carried out, cells expressing NTD showed hyper-sensitivity (Fig. 9C) to UV light – a typical characteristic of NER deficiency, indicating that NTD expression could influence the NER pathway.

Alterations in the levels of NER components or Mfd have an effect on generation of spontaneous mutations [25], [26]. To analyze the frequency of spontaneous mutations, mutator assays were carried out using *E. coli* cells expressing NTD of MtbMfd and *mfd* deficient strain (UNCNOMFD) and mutation frequencies were calculated as described in Materials and Methods. The reduction in mutation frequency in NTD expressing cells (0.32) and UNCNOMFD (0.72) compared to wild-type (1) (Fig. 9D) indeed supports the dominant negative effect of NTD on NER.

## Discussion

Every genome invests significant effort in ensuring genomic integrity and stability. A plethora of repair mechanisms which exist in the organisms, functionally cooperate to safeguard the genomes by repairing the diverse range of damages inflicted on the DNA. Most bacterial genomes harbor a full arsenal of repair pathways viz photoreactivation, base excision repair, nucleotide excision repair, mismatch repair, recombination repair and SOS response. Sequencing of *M. tuberculosis* genome facilitated the identification of the various repair processes operational in mycobacteria. Although several DNA repair pathways were found in mycobacteria, surprisingly mismatch repair genes were absent. However, the components needed for NER and transcription coupled repair were present. In this study, we have carried out detailed characterization of MtbMfd. At first glance, the primary sequence analysis of MtbMfd revealed significant similarity to EcoMfd with respect to size, domainal organization and conservation of the motifs. Thus, as one could predict, the purified MtbMfd protein had typical activities of Mfd viz DNA binding, ATPase and translocase. Moreover, the Mtb*mfd* complemented *mfd* deficiency in an *E. coli* strain in two different assays viz UV survival and road-block repair (Figs. 1 and 2). Thus, although overall similarity is about 38%, the Mfd function seems to be functionally conserved across these two widely divergent species. However, most surprisingly, the MtbMfd was found to occur in an oligomeric form in contrast to the monomeric form of EcoMfd (Figs. 4 and 5). The MtbMfd existed in both monomeric and hexameric form and deletion of the extreme C-terminus resulted in shifting of the equilibrium mostly towards the monomeric form. The higher stability of the hexameric form under various conditions as well as resistance to limited proteolysis suggests functional importance for the oligomeric MtbMfd (Fig. 6).

What could be the physiological significance of hexameric MtbMfd? This has turned out to be a very challenging question as both forms are found both *in vivo* and *in vitro* (Figs. 4 and 5). Since EcoMfd was found only in monomeric form, the monomer of MtbMfd could be the active form considering the similar domainal architecture. If so, hexameric form of MtbMfd could be non functional. However, the hexameric form was found to have ATPase activity (Fig. S3). Next, the possibility of ligand mediated transition in the protein as a mechanism for multimerization was considered. For instance, changes in the conformation stabilizes the oligomeric form of prokaryotic enhancer binding protein, NtrC1; binding of ATP analogues stabilize oligomeric form of the protein and facilitate its binding to Sigma 54 [27]. Further, the McrBC restriction endonuclease assembles into a ring structure in the presence of GTP and its analogues [28]. However, in case of MtbMfd, the presence of DNA or ATP did not alter the oligomerization status indicating that it is rather an intrinsic property of the protein (Fig. S4 D & E). The increased sensitivity of the monomer and the relative stability of the hexamer to protease digestion (Fig. 6A & B) suggested that the hexamer was a stable form of MtbMfd probably serving as a reservoir inside the cell for its ready availability at the repair site during transcription. Intracellular factors may trigger the monomerization of the protein prior to its action at the stalled transcription site. One scenario that could be envisioned is that upon encountering DNA damage, the hexameric MtbMfd is available for immediate recruitment to the stalled transcription complex.

The studies described here allow us to compare the properties of MtbMfd with very well studied EcoMfd. In addition to the differences in oligomerization properties described above, the enzymes seem to differ significantly in a few other properties. While the full length proteins do not reveal significant differences in their catalytic properties, the CTDs show vast differences in their translocase activities, which correlate well with their respective ATPase activities (Table 2). This would mean that the sequences in CTD are responsible for the distinct properties of the two Mfds with respect to oligomerization potential and control of translocase activity. One important finding of the present study is the binding of ATP to NTD of MtbMfd. All Mfd NTDs resemble UvrB and possess the degenerate ATPase motifs. Indeed, on the basis of sequence and structural similarities, it has been suggested that Mfds have evolved from UvrB incorporating an additional translocase activity [18]. UvrB has cryptic ATPase activity while the NTD of Mfd may have lost the activity as it possesses degenerate Walker motifs. Structural analysis of EcoMfd and ATPase assays revealed that NTD of EcoMfd lacks functional ATP binding sites [18]. In contrast, NTD of MtbMfd binds ATP but is hydrolysis deficient (Fig. 7). A closer comparison of the amino acid sequences in the Walker A motif reveal that conserved lysine 45 of UvrB has been replaced by arginine in case of NTD of MtbMfd. It has been shown previously that mutation of lysine 45 to alanine, aspartate and arginine led to a loss of ATPase activity of UvrB [29]. Thus, MtbMfd seems to be a natural mutant of UvrB. *B. subtilis* Mfd also has arginine in this location while EcoMfd has cysteine [18].These differences could account for the observed difference in ATP binding in case of EcoMfd and MtbMfd. Single amino acid change appears to be one of the determinants in evolving an ATPase deficient NTD from ATP hydrolyzing UvrB although other residues in both Walker A and B motifs could also contribute to the loss of function. What could be the physiological basis of ATP binding to the NTD of MtbMfd? It could serve as an ATP reservoir for ATPase/translocase activity. Alternatively, it may have a role in altering the stability or conformation of the protein or may be just vestigial. The C-terminal domain of Mfd has dedicated ATPase and the role of the NTD appears to be only in UvrA recruitment. Having ATPase activity at NTD may interfere with the recruitment process and the loss of ATPase activity in evolving Mfd seems to be crucial for its function. The role in UvrA recruitment is amply evident from the dominant negative effect of NTD expression on NER pathway.

Analysis of sequenced bacterial genomes revealed that Mfd is found in most of the genomes highlighting the importance of transcription coupled DNA repair for ensuring error free gene expression. In spite of this evolutionary conservation and the high degree of relatedness, the present findings reveal differences in Mfd between the organisms. These differences may indicate the appropriate tailoring of the functions based on the nature of the genome (size, G+C content) and the transcription process. The high G+C content of mycobacterial genome [30], presence of a large number of sigma factors [31], varied promoter architecture [32] and slow rate of transcription [33], [34] hint at some distinctive role of Mfd in *M. tuberculosis*. Thus, the differences in the properties of MtbMfd could be due to mycobacteria specific optimization in its function. All mycobacterial genomes sequenced have Mfd, highly homologous to that of MtbMfd and hence, are likely to display similar properties. Moreover, the gene is located at a fixed location between TetR and MazG regulatory proteins in most of the mycobacterial genomes indicating its early existence in the genus pointing at its crucial intracellular role.

## Materials and Methods

### Bacterial strains and plasmids

*Mycobacterium tuberculosis* H37Rv (*M. tuberculosis*) strain was used as a source of genomic DNA for polymerase chain reaction (PCR) to amplify *mfd* gene. AB1157 (*thr leu6 thi1 lacY1 galK2 ara14 xyl5 mtl1 kdgK51 proA2 his4 argE3 str31 tsx33 supE44*) and its derivative UNCNOMFD (same as AB1157 but *mfd*Δ, kan^r^) was used for complementation studies [8].

*E. coli* strain DH5α (F' *endA1 hsd R17* (rk^−^ mk^−^) *glnV44 thi1 recA1 gyrA* (Nalr) *relA1*Δ (*lacIZYA* – *argF*) *U169 deoR* (φ80d *lac*Δ (*lac*ZM15)) was obtained from New England Biolabs and used for transformation and purification of plasmid DNA. BL21 (DE3) pLysS (F^−^ *ompT hsdSB* (rB^−^ mB^−^) *gal dcm* (DE3) pLysS (Cam^r^) and its derivatives TUNER and ER2655 were used for overexpression and purification of proteins.

pET32a, pET28a, pET21a, pET14b (Novagen) pRSETA (Invitrogen) and pTrc99A (New England Biolabs) plasmids were used for cloning of *Mtbmfd* gene and its derivatives. Purification of plasmid DNA was carried out by alkaline lysis method [35].

### Chemicals, oligonucleotides and radiolabeling

Restriction endonucleases, T4 DNA ligase, T4 polynucleotide kinase were obtained from New England Biolabs. Ampicillin, kanamycin, tetracycline, streptomycin, proteinase K, trypsin, V8 protease, protease inhibitor cocktail, Coomassie brilliant blue (R-250), IPTG were obtained from Sigma (USA). ^14^C chloramphenicol (57.0 mCi/mmol) and ^32^P-ATP (3500 Ci/mmol) were procured from GE healthcare (Uppsala, Sweden) and BRIT India respectively. All other reagents used were ultra pure, analytical or molecular biology grade.

Oligonucleotides used in this study were synthesized by Sigma Genosys, and their sequences given in Table 1. Oligonucleotides were labeled at the 5′-end with [γ-^32^P] ATP (20 µCi) using T4 polynucleotide kinase. The labeled oligonucleotides were purified by nucleotide removal kit (Qiagen, USA) and DNA was further purified from native polyacrylamide gels.

### Amplification and cloning of *M. tuberculosis mfd* (Mtb*mfd*)

The ∼3.7 kb long Mtb*mfd* was amplified in three different fragments F1 (1.3 kb), F2 (1.5 kb) and F3 (0.9 kb) by PCR. *M. tuberculosis H37Rv* genomic DNA was taken as a template and primer pairs used were PF1-PR1, PF2-PR2 and PF3-PR3 respectively (Table 1). The primers were designed based on the annotated complete genome sequence of *M. tuberculosis* [30]. All three fragments were cloned sequentially into pET32a vector to obtain full length product without altering the nucleotide sequence of Mtb*mfd* gene. The full length Mtb*mfd* was sub-cloned into the bacterial expression vector pET28a (pETmfd) and pTRc99A (pTrcmfd) using the restriction enzyme sites NdeI-HindIII and BamHI-HindIII respectively. A construct containing the NTD of Mtb*mfd* was generated by cloning the F1 fragment into pET14b using NdeI-KpnI sites. CTD was generated by releasing 1.9 kb fragment of Mtb*mfd* gene with EheI-HindIII enzymes and further cloned into pRSETA vector for expression. A MtbMfd having C-terminus deletion (184 aa) was generated using specific primer set PF3-PR3Δ (Table 1) and replaced in place of wild type F3 fragment in pETmfd clone. MfdD778A having point mutation in Walker B motif of the ATPase domain was generated by mega primer method, using specific primer set WbF-WbM whereas WbF-WbR primer set was used for screening and confirmation of mutation.

### UV-survival assay

Wild-type *E. coli* (AB1157) was used as a control and *mfd* deficient *E. coli* strain (UNCNOMFD) was transformed with either pTrcmfd construct containing *Mycobacterium mfd* or pTrc99A vector alone. An overnight grown culture was inoculated into fresh LB medium and grown to 0.6 OD_600 nm_. 10 ml of each culture were pelleted down, dissolved in half the volume of ice cold normal saline (0.9% NaCl) and irradiated with UV light (1 J/m^2^ flux) for different time points. Different dilutions of each culture (± UV) were plated on LB agar with appropriate antibiotics. Twelve hours later, colonies were counted and survival (S/S_0_) was measured and plotted against time. All the procedures after UV irradiation were carried out in dark.

To analyze the effect of NTD expression on UV survival, similar methodology was used with minor modification. After UV irradiation different dilutions of culture (1 µl) were spotted on agar plate containing 100 µg/ml ampicillin and 35 µg/ml choramphenicol and incubated overnight at 37°C. Formation of colonies were observed and documented.

### Road-block reporter assay

AB1157 or UNCNOMFD cells were transformed with pRCBCAT1 [20] construct along with pTrcmfd or pTrc99A, grown at 37°C in 2XYT medium. After cells were grown to 0.6 OD_600 nm_, 3 ml of cultures were harvested by centrifugation and cells lysed with 180 µl of TME buffer (25 mM Tris-Cl pH 8.0, 2 mM β-mercaptoethanol and 1 mM EDTA) supplemented with 20 µl of 1 mg/ml lysozyme and 6 µl of 1 mg/ml DNaseI. The mixture was incubated for 5 minutes at room temperature followed by freeze thawing in liquid nitrogen and spun at 12,000 rpm for 15 minutes. Supernatants were stored in equal amount of storage buffer (20 mM Tris-Cl pH 8.0, 200 mM NaCl, 20 mM mercaptoethanol and 80% glycerol) and snap frozen at −80°C. CAT activity was measured in Tris-Cl buffer pH 8.0 containing 3 mg/ml acetyl coA, 500 ng of protein, 10 mM chloramphenicol (radiolabeled ^14^chloramphenicol was used as tracer) at 37°C for 30 minutes and stopped by the addition of 500 µl of ethyl acetate. The ethyl acetate phase was separated in fresh tube, dried in speed vac and 5 µl was loaded onto silica plate. The plate was developed in a chamber saturated with chloroform: methanol (95∶5) and exposed to phosphor Imager and quantified by image gauge software. CAT activities are expressed as nmol of chloramphenicol acetylated/min/mg of protein.

### Purification of MtbMfd

*E. coli* BL21 (DE3) pLysS TUNER cells harboring the pETmfd construct were grown at 37°C in TB broth containing 30 µg/ml kanamycin and 35 µg/ml chloramphenicol to an 0.6 OD_600_, and induced by the addition of 0.3 mM IPTG. After 10 hrs of incubation at 18°C, bacterial cells were harvested by centrifugation at 8,000 rpm for 10 min, the pellet was resuspended in buffer A (50 mM Tris-Cl pH 8.0, 500 mM NaCl, 5 mM imidazole, 10% glycerol, 10 mM β-mercaptoethanol and 0.01% triton X)) supplemented with 1 mM PMSF and EDTA-free protease-inhibitor cocktail (Sigma) and lysed by sonication. For purification, cell free lysate was obtained by centrifugation of at 20,000 rpm for 1 hr. Lysate was loaded onto a Ni^2+^-NTA column (Amersham Biosciences) and eluted using a 15–300 mM imidazole linear gradient. Fractions containing MtbMfd were pooled and dialyzed against buffer B (20 mM Tris-Cl pH 8.0, 100 mM NaCl, 10% glycerol, 1 mM EDTA and 10 mM β-mercaptoethanol). MtbMfd containing fractions were further purified on a Heparin-sepharose column (Amersham Biosciences) using a linear gradient from 100–400 mM NaCl and finally by size-exclusion chromatography on a Superdex 200 column (Amersham Biosciences) in a buffer B consisting 500 mM NaCl. The purity of the protein was analyzed on SDS-PAGE and by silver-staining and concentration of protein was determined by measuring OD at 280 nm as well as by Bradford method [36]. Variants of MtbMfd protein were essentially purified using the same protocol. For NTD purification Q-sepharose column was used instead of heparin-sepharose.

### Generation of polyclonal antibodies against MtbMfd

Antibodies were raised in rabbit by injecting 500 µg of denatured MtbMfd (native protein) with an equal volume of Fruend's complete adjuvant subcutaneously. ∼5 ml of pre-immune serum was collected on day 1 prior to injection. Approximately 300 µg of protein with Fruend's incomplete adjuvant was injected after three weeks (21 days) as first booster dose. The second booster dose was given similarly with 300 µg of protein after 15 days interval after the first booster dose. After 7 days of second booster, the rabbit was bled, blood was collected, centrifuged and serum stored in aliquots at −20°C.

### Western blot analysis

For Western blot analysis, *E. coli* lysate or purified recombinant proteins were subjected to 10% SDS-PAGE containing 0.1% SDS after solubilization with buffer B. Proteins were transferred on to a nitrocellulose membrane Hybond-C for 2 hr at 200 mA in transfer buffer (39 mM glycine, 48 mM Tris, 0.037% SDS, and 20% methanol). After the transfer, the membrane was blocked with blocking solution. The membrane was immuno stained with polyclonal rabbit anti-MtbMfd antibodies (1∶10000 dilutions) for 2–3 hr at room temperature followed by three times washing with PBST (10 min each). The membrane was further stained with secondary antibody, anti rabbit IgG tagged with HRP for one hour followed by washing for three times with PBST [35]. The blot was developed using ECL kit (GE, Amersham).

### Size exclusion chromatography

Native molecular mass of MtbMfd and its variants was determined by gel filtration chromatography. Superose 6 column was equilibrated in buffer B. The void volume (*V*_o_) of the column was determined using blue dextran (2000 kDa) and was found to be 7.5 ml and the bed volume 24 ml. The column was calibrated with suitable molecular weight markers ranging from 66 kDa to 669 kDa; thyroglobulin (669 kDa), ferritin (440 kDa), aldolase (150 kDa) and bovine serum albumin (66 kDa). Fractions (0.5 ml each) were collected and the presence of protein was confirmed by SDS-PAGE and by Bradford's method [36]. The elution volumes (*V*_e_) of marker proteins and wild-type or mutant MtbMfd were determined. The molecular mass was calculated from plot of *V*_e_/*V*_o_ versus log molecular weight. The molecular weights corresponding to the peaks of MtbMfd and its derivatives were calculated from the standards graph using graph pad prism software.

### Glutaraldehyde cross-linking

MtbMfd (4 µg/reaction) was incubated with increasing amounts of glutaraldehyde to a final concentration range of 0.01–0.1%, on ice for 20 minutes. Reactions were stopped by adding SDS gel loading dye and products were separated on a 4.5% denaturing polyacrylamide gel by electrophoresis and visualized by silver-staining.

### Growth and preparation of *M. tuberculosis H37Ra* cell extract

*M. tuberculosis H37Ra* strain was grown in 7H9 medium (Difco, BD, USA) containing 10% ADC supplements (for 1 liter; 8.5 g NaCl, 50 g BSA, 20 g glucose and 0.03 g Catalase) and 0.05% Tween 80 to 0.7 OD. Cells were harvested and pelleted by centrifugation at 8, 000 rpm at 4°C. Crude cell lysate was prepared in Tris buffer pH 8.0 (20 mM Tris-Cl, 100 mM NaCl, 10% glycerol, 0.1 mM EDTA and 5 mM β-mercaptoethanol) by sonication followed by ultracentrifugation at 100,000 g (S100) for 3 hrs at 4°C. The supernatant was used for native-PAGE Western and gel filtration analysis for Figure 5.

### Non-denaturing PAGE (native PAGE)

Polyacrylamide gel (6%) was pre run at 100 V for 1 hr at room temperature in 1× buffer (Tris/glycine buffer, pH 8.3, containing 12.5 mM Tris-HCl and 125 mM glycine). Samples were mixed with 1× loading dye (without SDS) and were run for 6 hrs at room temperature. For Western blot experiments, proteins after electrophoresis were transferred to Hybond C membrane at room temperature at 100 mA for 10 hrs in a transfer buffer followed by probing with anti-MtbMfd antibody as described earlier.

### Dynamic light scattering

Dynamic light scattering (DLS) experiments were performed on a DynaPro Molecular Sizing Instrument (Protein Solutions). DLS measures fluctuations in the intensity of light scattered by a macromolecular solution which is related to its hydrodynamic Radius (R_H_). Purified His-tagged MtbMfd was dialyzed in filtered 10 mM Tris-HCl, pH 8.0 buffer and centrifuged at 18,000 rpm for 30 min and loaded into a quartz cuvette before measurement. Several measurements were taken at 25°C and analyzed using DYNAMICS Version 3.30 software (Protein Solutions). Data collection times of 10 s were used in all the cases, for a minimum of 15 acquisitions.

### Limited Proteolysis

Monomer and hexamer fractions of MtbMfd (4 µg/reaction) obtained by gel filtration chromatography were subjected to protease digestions at 25°C by trypsin or *Staphylococcus* V8 protease. Trypsin∶MtbMfd ratio was 1∶100 and V8∶MtbMfd ratio was 1∶200 per reaction. At various time points, aliquots were removed and PMSF was added to stop the reaction. SDS-sample buffer was added followed by boiling at 95°C for 5 minutes. The samples were analyzed on 10% polyacrylamide gels containing 0.1% SDS and visualized either by Commassie brilliant blue or silver staining.

### Analysis of NTD-ATP interaction by Fluorescence Spectroscopy

Fluorescence emission spectra were measured for NTD of MtbMfd on a Shimadzu, RF 5000 spectrofluorimeter using a 1-cm quartz cuvette at 25°C. The emission spectra were recorded over a wavelength of 300–400 nm with an excitation wavelength of 280 nm. NTD was allowed to equilibrate for 2 min in ATPase buffer (without MgCl_2_) before measurements were made. Small aliquots of ATP (final concentration 100 µM-1 mM) were added to NTD (1 µM) before recording the spectra. The binding of ATP to proteins resulted in quenching of tryptophan fluorescence. The slit widths of 10 nm for excitation and emission were used and each spectrum recorded was an average of three scans. Data analyzed according to Stern-Volmer relationship which is represented by
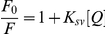
where *F_0_* and *F* are fluorescent intensities in the absence and presence of ATP respectively, *K_SV_* is the Stern-Volmer constant and Q is the quencher (ATP) concentration [37].

### ATPase assay

ATPase activity of MtbMfd and its mutants were assayed as described previously [38] with minor modifications in 10 µl of buffer containing 40 mM HEPES pH 8.0, 50 mM KCl, 5 mM DTT, 8 mM MgCl_2_ and 2 mM ATP, 100 µg of bovine serum albumin per ml, 4% glycerol and 6% polyethylene glycol 6000. pUC 19 DNA (1 µM) was used in the reaction mixture unless otherwise specified, MtbMfd and its mutant proteins were included at a concentration of 250 nM and [γ^32^P] ATP was used as a tracer. Reactions were carried out at 37°C for 30 minutes, terminated by the addition of 2 µl of 50 mM EDTA and 0.5 µl of each reaction mix was spotted on a PEI-Cellulose TLC sheet. TLC sheet were developed in 1.2 M LiCl and 0.1 mM EDTA, exposed to Fuji BAS phosphor screen, scanned using Fuji Phosphor Imager and quantified by image gauge software. All kinetic parameters were measured under steady state conditions (S>>E) using non linear regression analysis with the help of Prism v.5 software. All enzymatic assays were carried out at least three times.

### Translocase or TFO (triplex forming oligonucleotide) displacement assay

Translocase assays were carried out essentially as described previously [23] with minor modifications. A 72 mer oligonucleotide containing triplex forming region was cloned into pUC19 vector in EcoRI-HindIII sites. A 300 base pair fragment was released from pUC19 using PvuII enzyme. End labeled (^32^p-γATP) 22-mer oligonucletide was incubated with 300 mer dsDNA in MES buffer pH 5.5 containing 10 mM MgCl_2_ at 20°C overnight. The triplex formed was purified from native polyacrylamide gel and used as a substrate in the translocase assay. 250 nM of each protein (MtbMfd, CTD and MfdΔC) were separately incubated with triplex DNA in 50 mM Tris-Cl (pH 8.0) containing 10 mM MgCl_2_, 2 mM ATP and 1 mM DTT at 20°C and aliquots were taken at different time points. Reactions were stopped by adding GSMB buffer (15% glucose, 3% SDS, 250 mM MOPS pH 5.5 and 0.4 mg/ml bromophenol blue)) and separated on 5% native polyacrylamide gel in TAE buffer (pH 5.5) containing 5 mM MgCl_2_ and 5 mM sodium acetate at 4°C and visualized by Fuji Phosphor Imager. Quantitation was carried out using image gauge software.

### Growth curve analysis of NTD expressing *E. coli* cells

pET14b vector carrying NTD construct (pETNTD) was transformed in BL21 pLysS cells. Colonies were picked and grown overnight at 37°C. Cells transformed with empty vector were taken as control. The overnight grown culture was used as a primary inoculum (1%) and inoculated in to 100 ml of LB media containing 100 µg/ml ampicillin and 35 µg/ml chloramphenicol and allowed to grow at 37°C. Aliquots were taken at every 30 min time intervals and OD was monitored at 600 nm. Growth curves were obtained by plotting OD_6oo nm_ in Y-axis and time in X-axis.

For growth on the solid medium, equal number of cells from log phase cultures were taken from each sample and streaked on the agar plates having suitable antibiotics. Plates were incubated overnight at 37°C, formation of colonies was observed and documented.

### Determination of mutation frequencies for Rif^s^→Rif^r^ spontaneous mutations

The frequencies of rifampicin resistant mutants were determined by plating the overnight grown culture on plates containing ampicillin plus rifampicin (100 µg/ml). Duplicate samples were also plated on LB agar with ampicillin (100 µg/ml) to determine the cell viability. Plates were incubated at 37°C overnight. for scoring the rifampicin resistant colonies. Fold change was calculated by dividing the number of Rif^r^ colonies by that of total number of colonies [25].

## Supporting Information

Figure S1 **Gel filtration chromatography of MtbMfd under different conditions and their elution profiles.** **A**. Profile of MtbMfd and Thyroglobulin, when both the proteins were co-injected into the column. 1 & 2 represents peak 1 & peak 2 and their retention volume are indicated on top of the peaks. The panel below shows the SDS-PAGE profile of the same, where peak 1 retains both the proteins and peak 2 contains only MtbMfd. **B**. MtbMfd (250 µg). **C**. MtbMfd (700 µg). **D**. MtbMfd (700 µg) in presence of 500 mM NaCl. Retention volumes are indicated on the top of the respective peaks; where 7.5 ml corresponds to void volume of the column; 11.3 ml is the retention volume of hexameric species and 14.6 ml is the retention volume of the monomeric species of the MtbMfd.
(TIF)

Figure S2 **Gel filtration chromatography of crude cell lysate of** ***M. tuberculosis Ra*** **and native MtbMfd.** **A**. Elution profile of total proteins present in crude cell lysate of *M. tuberculosis Ra*. **B**. Elution profile of purified untagged or native MtbMfd (400 µg), peak 1 & 2 corresponding to hexamer and monomer of MtbMfd respectively. Retention volumes of respective peaks are indicated on the top of the each peak. Peak at 7.5 ml corresponds to void volume of the column.
(TIF)

Figure S3 **Comparison of ATPase activity of hexamer and monomer of MtbMfd.** Hexamer and monomer fractions of MtbMfd was separated by gel filtration chromatography and subjected to ATPase assay, data was quantified by image gauze software. Specific ATPase activity was expressed as pmoles ATP hydrolyzed per min per µg of protein.
(TIF)

Figure S4 **Gel filtration chromatography of MtbMfd in presence of ATP and DNA.** 700 µg of MtbMfd was incubated with excess of ATPγS or DNA or both ATPγS and DNA for 30 min on ice and then co-injected into the column. Elution profiles were then monitored. **A**. MtbMfd alone. **B**. ATPγS. **C**. pUC19 DNA. **D**. MtbMfd+ATPγS **E**. MtbMfd+ATPγS+pUCDNA. Retention volumes are indicated on top of the respective peaks.
(TIF)
