# Supplementary figures and images for: Distinct Properties of Hexameric but Functionally Conserved Mycobacterium tuberculosis Transcription-Repair Coupling Factor
Source: PLoS One. 2011 Apr 29;6(4):e19131. doi: 10.1371/journal.pone.0019131 (PMC3084762; doi:10.1371/journal.pone.0019131)

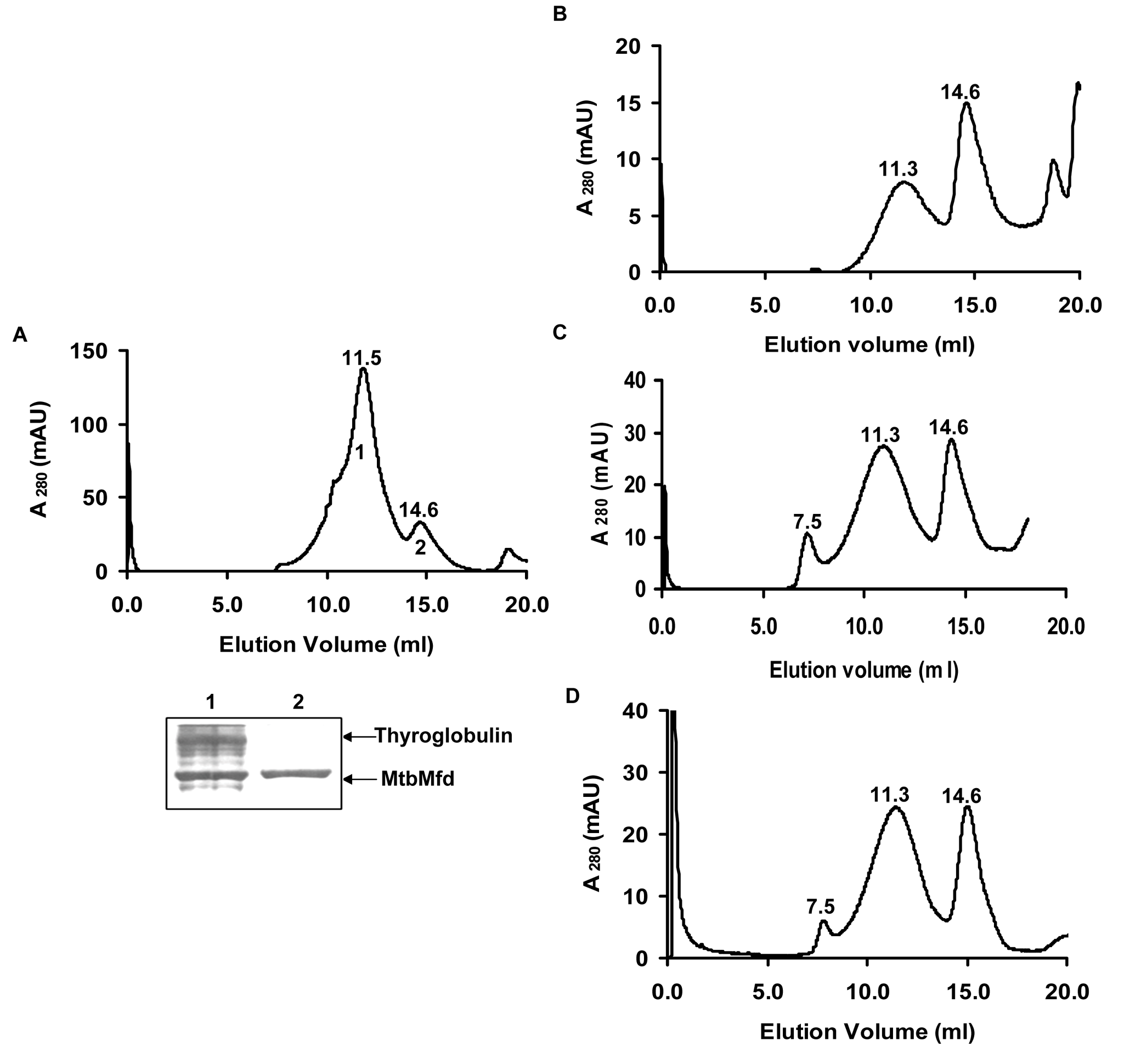

Supplement: Figure S1 — Gel filtration chromatography of MtbMfd under different conditions and their elution profiles. A. Profile of MtbMfd and Thyroglobulin, when both the proteins were co-injected into the column. 1 & 2 represents peak 1 & peak 2 and their retention volume are indicated on top of the peaks. The panel below shows the SDS-PAGE profile of the same, where peak 1 retains both the proteins and peak 2 contains only MtbMfd. B. MtbMfd (250 µg). C. MtbMfd (700 µg). D. MtbMfd (700 µg) in presence of 500 mM NaCl. Retention volumes are indicated on the top of the respective peaks; where 7.5 ml corresponds to void volume of the column; 11.3 ml is the retention volume of hexameric species and 14.6 ml is the retention volume of the monomeric species of the MtbMfd. (TIF) [file pone.0019131.s001.tif]

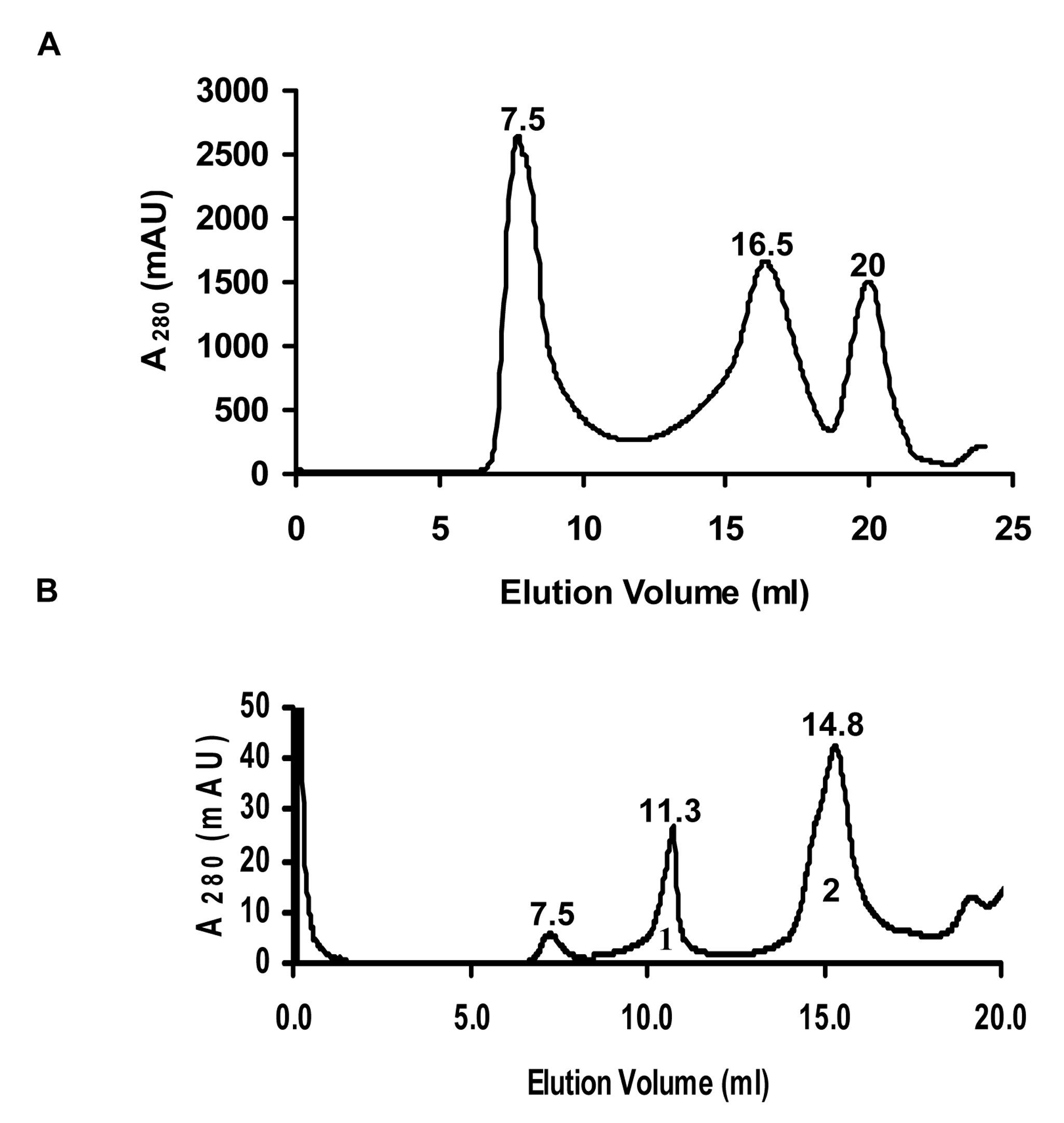

Supplement: Figure S2 — Gel filtration chromatography of crude cell lysate of M. tuberculosis Ra and native MtbMfd. A. Elution profile of total proteins present in crude cell lysate of M. tuberculosis Ra. B. Elution profile of purified untagged or native MtbMfd (400 µg), peak 1 & 2 corresponding to hexamer and monomer of MtbMfd respectively. Retention volumes of respective peaks are indicated on the top of the each peak. Peak at 7.5 ml corresponds to void volume of the column. (TIF) [file pone.0019131.s002.tif]

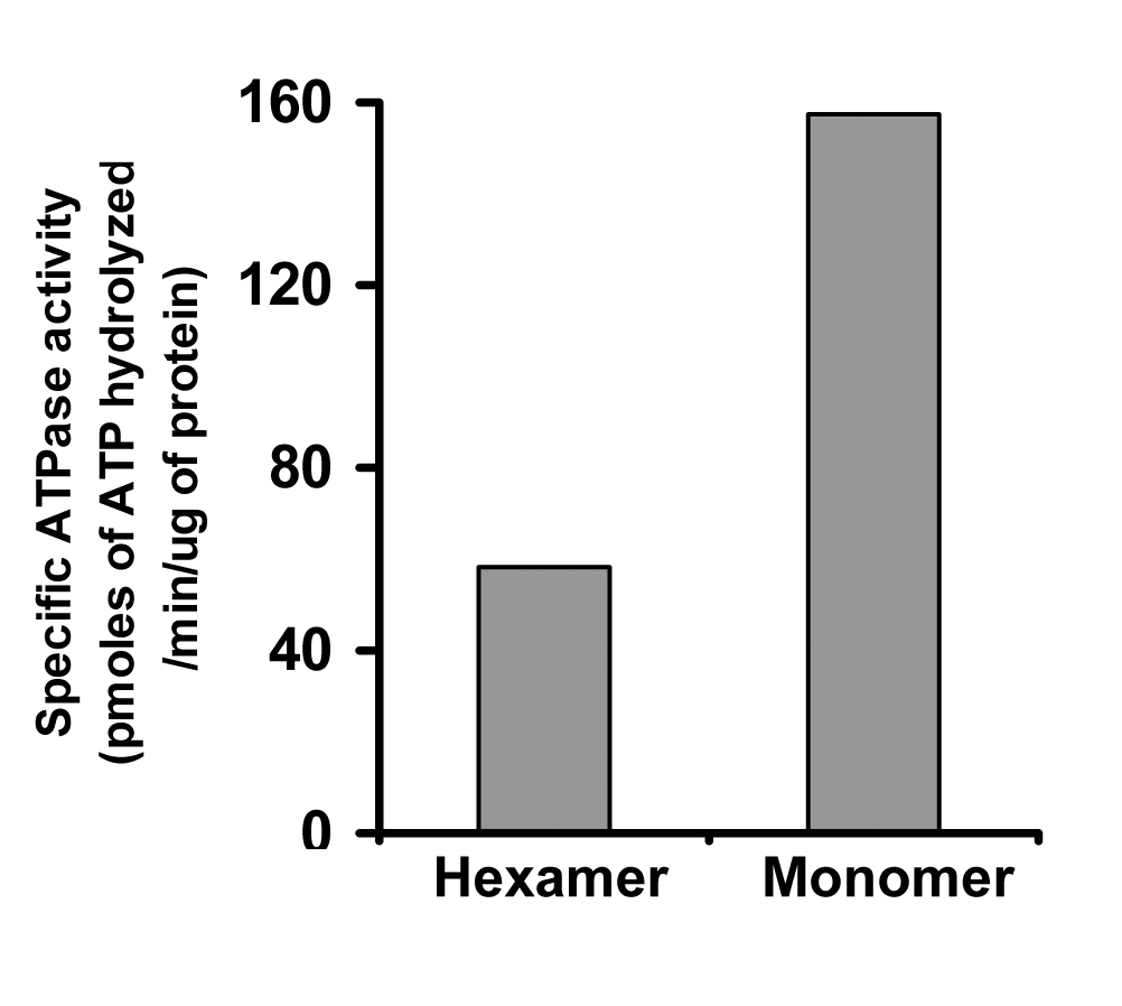

Supplement: Figure S3 — Comparison of ATPase activity of hexamer and monomer of MtbMfd. Hexamer and monomer fractions of MtbMfd was separated by gel filtration chromatography and subjected to ATPase assay, data was quantified by image gauze software. Specific ATPase activity was expressed as pmoles ATP hydrolyzed per min per µg of protein. (TIF) [file pone.0019131.s003.tif]

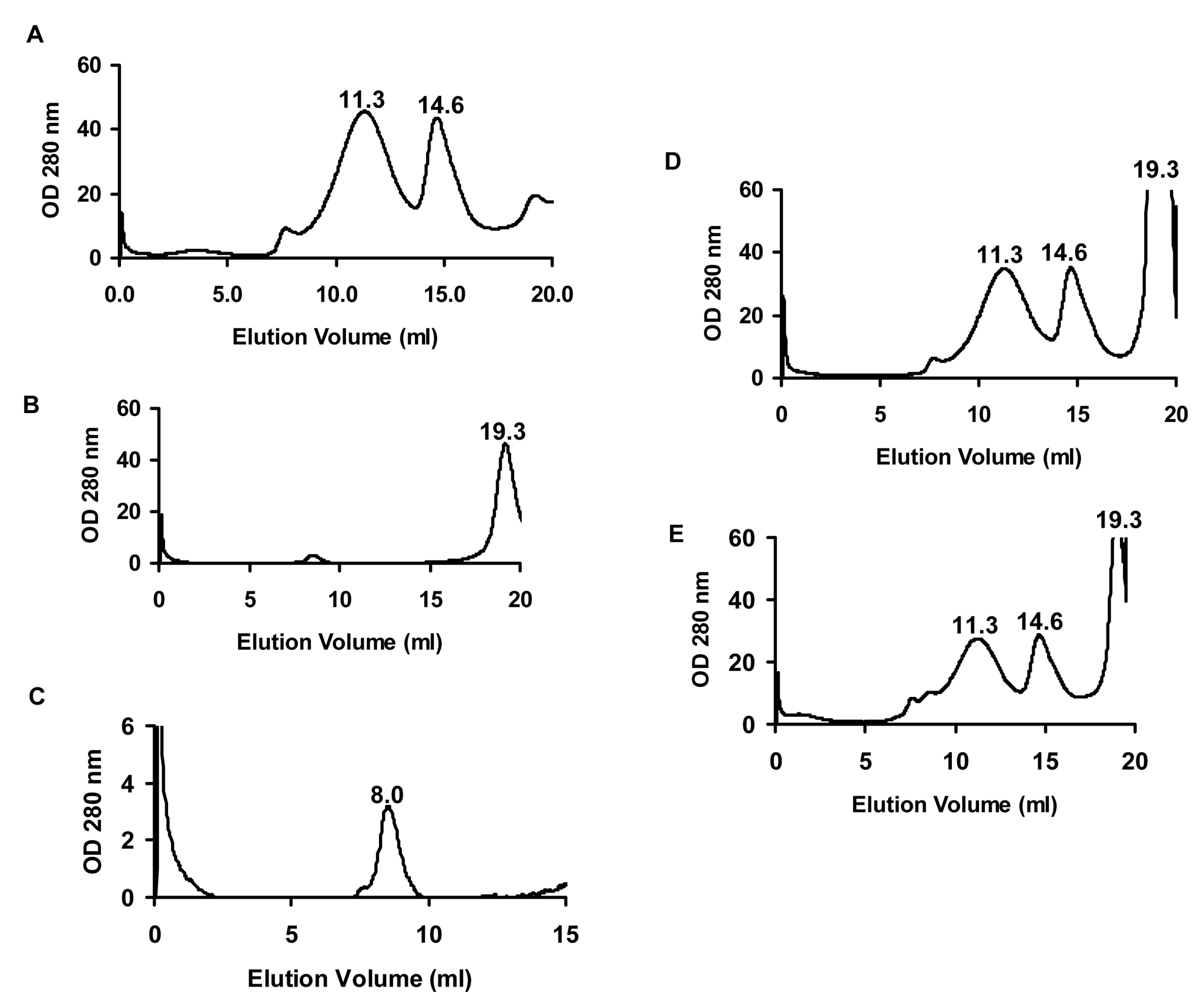

Supplement: Figure S4 — Gel filtration chromatography of MtbMfd in presence of ATP and DNA. 700 µg of MtbMfd was incubated with excess of ATPγS or DNA or both ATPγS and DNA for 30 min on ice and then co-injected into the column. Elution profiles were then monitored. A. MtbMfd alone. B. ATPγS. C. pUC19 DNA. D. MtbMfd+ATPγS E. MtbMfd+ATPγS+pUCDNA. Retention volumes are indicated on top of the respective peaks. (TIF) [file pone.0019131.s004.tif]
